# Crystal Structure of an Archaeal Tyrosyl-tRNA Synthetase Bound to Photocaged L-Tyrosine and Its Potential Application to Time-Resolved X-ray Crystallography

**DOI:** 10.3390/ijms231810399

**Published:** 2022-09-08

**Authors:** Toshiaki Hosaka, Kazushige Katsura, Yoshiko Ishizuka-Katsura, Kazuharu Hanada, Kaori Ito, Yuri Tomabechi, Mio Inoue, Ryogo Akasaka, Chie Takemoto, Mikako Shirouzu

**Affiliations:** Laboratory for Protein Functional and Structural Biology, RIKEN Center for Biosystems Dynamics Research, 1-7-22 Suehiro-cho, Tsurumi-ku, Yokohama 230-0045, Kanagawa, Japan

**Keywords:** nonnatural amino acid, cell-free protein synthesis, X-ray crystallography, caged amino acid, *O*-(2-nitrobenzyl)-L-tyrosine

## Abstract

Genetically encoded caged amino acids can be used to control the dynamics of protein activities and cellular localization in response to external cues. In the present study, we revealed the structural basis for the recognition of *O*-(2-nitrobenzyl)-L-tyrosine (*o*NBTyr) by its specific variant of *Methanocaldococcus jannaschii* tyrosyl-tRNA synthetase (*o*NBTyrRS), and then demonstrated its potential availability for time-resolved X-ray crystallography. The substrate-bound crystal structure of *o*NBTyrRS at a 2.79 Å resolution indicated that the replacement of tyrosine and leucine at positions 32 and 65 by glycine (Tyr32Gly and Leu65Gly, respectively) and Asp158Ser created sufficient space for entry of the bulky substitute into the amino acid binding pocket, while Glu in place of Leu162 formed a hydrogen bond with the nitro moiety of *o*NBTyr. We also produced an *o*NBTyr-containing lysozyme through a cell-free protein synthesis system derived from the *Escherichia coli* B95. ΔA strain with the UAG codon reassigned to the nonnatural amino acid. Another crystallographic study of the caged protein showed that the site-specifically incorporated *o*NBTyr was degraded to tyrosine by light irradiation of the crystals. Thus, cell-free protein synthesis of caged proteins with *o*NBTyr could facilitate time-resolved structural analysis of proteins, including medically important membrane proteins.

## 1. Introduction

Photoresponsive caged compounds are useful tools for investigating cellular pathways, biochemical reactions, and enzymatic processes in time-resolved manners [1,2]. The release of caged groups and their subsequent changes in target molecules trigger downstream events that can be analyzed in detailed timeframes [3,4]. The substrates/inhibitors of enzymes and the agonists/antagonists of signaling molecules (receptors and channels) are thus photomanipulated to trigger targeted events in cell signaling and neuronal activities to precisely elucidate downstream responses [5,6]. “Caged proteins” with such photoreactive groups are prepared using complicated methods which are considered a hurdle for applications [7]. In contrast, the genetic encoding of caged amino acids offers a more facile and robust approach for biosynthesizing the photoresponsive proteins. These synthetic amino acids are recognized by specific variants of aminoacyl-tRNA synthetase and attach to tRNA, which in turn translates codons (mostly the UAG stop codon) assigned to the amino acids. The molecular basis for the specific binding of a caged amino acid (o-nitrobenzyl-3,4-dihydroxyphenylalanine, *m*-*o*NB-DOPA) has been studied using X-ray crystallography on a variant of *Methanocaldococcus jannaschii* tyrosyl-tRNA synthetase (*Mj*TyrRS)—designated *m*-*o*NBDOPARS—complexed with the substrate [8]. The crystal structure reveals how mutations remodeled the amino acid binding pocket of *m*-*o*NBDOPARS to accommodate the tyrosine derivative within a bulky caged group.

In the present study, we determined the crystal structure of another archaeal tyrosyl-tRNA synthetase variant (*o*NBTyrRS) complexed with *O*-(2-nitrobenzyl)-L-tyrosine. This amino acid, *o*NBTyr, has the same caged group as *m-o*NB-DOPA at a different position on the tyrosine ring [8]. The determined structure not only provides a precise picture of the *o*NBTyr recognition in terms of its specific variant, but also an interesting comparison between two different synthetases accommodating the same caged group. We also conducted a large-scale preparation of caged proteins and demonstrated the potential applicability of *o*NBTyr in time-resolved X-ray crystallography. We showed that this amino acid can be efficiently converted into a tyrosine residue within crystals, although the same amino acid is difficult to decage from other enzymes in the solution [9].

## 2. Results and Discussion

### 2.1. X-ray Crystallographic Study on oNBTyrRS Complexed with oNBTyr

**Figure 1:**
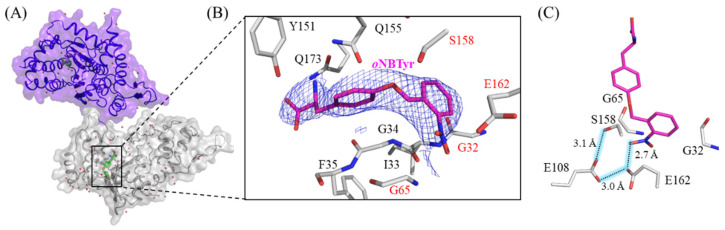
The crystal structure of *M. jannaschii o*NBTyrRS (Y32G, L65G, F108E, D158S, L162E, and D286R) complexed with *o*NBTyr. (**A**) Dimeric structure of *M. jannaschii o*NBTyrRS bound to *o*NBTyr in the asymmetric unit. The substrate *o*NBTyr and water molecules are shown by green sticks and red spheres. (**B**) Close-up view of *o*NBTyr. The |2F_o_ − F_c_| electron density map (contoured at 1.0 σ, blue mesh) corresponds to *o*NBTyr in the amino-acid binding site of *o*NBTyrRS. Mutated residues are indicated by the red letters. (**C**) Hydrogen-bonding interactions to recognize the *o*-nitrobenzyl group by *o*NBTyrRS. Numbers indicate the distance (Å) between two atoms connected by dashed lines.

*M. jannaschii* TyrRS (*Mj*TyrRS) is one of the aminoacyl-tRNA synthetases commonly used to introduce nonnatural amino acids into proteins by mutating in the substrate recognition sites [10]. *o*NBTyrRS has five amino acid replacements (Tyr32Gly, Leu65Gly, Phe108Glu, Asp158Ser, and Leu162Glu) at the tyrosine binding pocket of the wild-type *Mj*TyrRS [6,9,11]. The remaining replacement, Asp286Arg, was introduced into the synthetase variant to increase the charging efficiency toward suppressor tRNA with the CUA anticodon [12]. Firstly, 17 mg of *o*NBTyrRS was prepared from a 1 L Escherichia coli cell culture, and then co-crystallization of *o*NBTyrRS (10 mg/mL) with *o*NBTyr (1.4 mg/mL) was carried out using the vapor diffusion method. The diffraction data collected from a single crystal were processed, and the structure of *o*NBTyrRS with *o*NBTyr was determined at 2.79 Å resolution (Figure 1A and Table S1). All 306 amino acid residues could be modeled thus, and *o*NBTyrRS formed a dimer in a similar manner to the wild-type *Mj*TyrRS (PDB:1J1U); the dimer form is reportedly biologically active, as a tRNA substrate binds to two molecules of *Mj*TyrRS [12]. The *o*NBTyr-bound *o*NBTyrRS is similar to *Mj*TyrRS complexed with tRNA and L-tyrosine in overall structure, subunit arrangement, and domain architecture; the superposition of the Cα atoms of *o*NBTyrRS with those of *Mj*TyrRS showed a low average RMSD (0.7 Å) [12,13,14,15]. The electron density in the |2F_o_ − F_c_| map at the substrate binding site of *o*NBTyrRS unambiguously fits into the chemical structure of *o*NBTyr (Figure 1B).

As shown in Figure 1C, the five mutated residues play essential roles in the recognition of the structural features of *o*NBTyr. The substitutions of Tyr32 and Leu65 for Gly and Asp158 for Ser created enough space to accommodate the bulky nitrobenzyl group. This observation corresponds to previous reports that substitutions involving Tyr32 and Asp158 are preferable for *Mj*TyrRS variants to recognize new substrates larger than L-tyrosine (The PDB IDs and references are listed in Table S2). In addition, glutamates replaced with Phe108 and Leu162 form a so-called carboxyl (Glu108)-carboxylate (Glu162) dimer motif [16] (Figure 1C). This is consistent with the pKa values estimated by the PROPKA program, which are 7.81 and 7.55 for Glu108 and 4.76 and 3.50 for Glu162 in the respective monomer of the dimer [17,18]. Furthermore, these residues, together with the oxygen of the nitro group, formed a hydrogen-bonding network, which was stabilized by the participation of Ser158. The nitro group was slightly bent with respect to the planarity of the benzyl group, thus the crucial recognition of the nitro group, which is important for substrate discrimination, was exhibited.

### 2.2. Structural Comparison with the Wild Type and the Other Mutant of MjTyrRS

**Figure 2:**
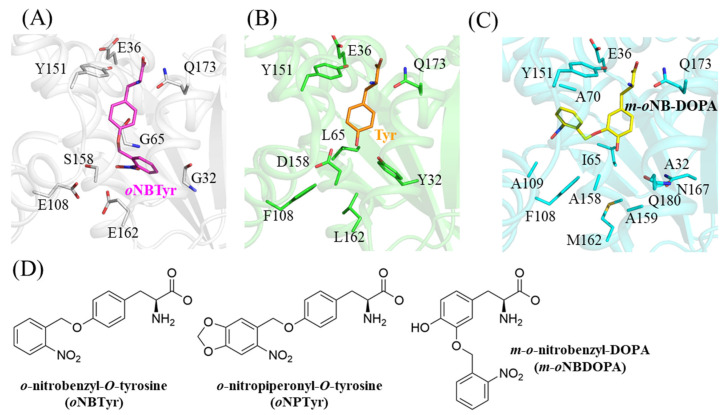
Structural comparison of the substrate binding modes of various *Mj*TyrRS. Structures of (**A**) *o*NBTyr in *o*NBTyrRS (this study); (**B**) tyrosine in the wild-type *Mj*TyrRS (PDB: 1J1U [12]); (**C**) *m-o*NBDOPA in the corresponding variant of *Mj*TyrRS (PDB: 5L7P [8]); (**D**) chemical structures of *o*NBTyr, *o*NPTyr, and *m*-*o*NB-DOPA.

The hydroxyl group of the tyrosine ring is recognized by the wild-type *Mj*TyrRS through hydrogen bonding interactions with Tyr32 and Asp158 (Figure 2B, PDB ID: 1J1U) [12]. The hydrophobic environment caused by Phe108 and Leu162 stabilizes these interactions. The replacements of these positions abolished this important mode of discrimination through direct interaction with the side chain of the substrate amino acid, and changed the hydrophobic environment into a hydrophilic one. Thus, these mutations properly switched the substrate specificity between the wild-type and variant synthetases. As this *o*NBTyrRS is reported to also recognize *o*-nitropiperonyl-O-tyrosine (*o*NPTyr), we modeled it into the binding pocket of *o*NBTyrRS by aligning the main chain of the substrate tyrosine [9,19]. Although the chemical structure of *o*NPTyr was larger than that of *o*NBTyr (Figure 2D), the fitting model showed no structural distortion (Supplementary Figure S1). This is because the substrate binding pocket of *o*NBTyrRS was open toward the surface of the enzyme.

As the other variant TyrRS for caged tyrosine, the crystal structure of *m*-*o*NBDOPARS bound to *m*-*o*NB-DOPA has been previously reported (PDB ID: 5L7P) [8]. The enzyme has 10 amino acid substitutions of the wild-type *Mj*TyrRS (Supplementary Table S2). The nitrobenzyl moiety of *m*-*o*NB-DOPA was attached to position 3 of the tyrosine ring and the hydroxyl group remains as tyrosine; however, the recognition mode varied from those of the wild-type or *o*NBTyrRS (Figure 2C). Three substituted residues (Tyr32Ala, Leu65Ile, and Asp158Ala) in *m*-*o*NBDOPARS form hydrophobic interactions together with Phe108, and the hydroxyl group of tyrosine is stabilized by van der Waals interactions with Ile65 and Ala158. This results in the position of tyrosine becoming similar to the wild-type *Mj*TyrRS. The nitrobenzyl group moiety is packed into a narrow hydrophobic space allowed by replacements with smaller amino acids at two positions (His70Ala and Gln109Ala) and is oriented to the interior of the enzyme, as opposed to *o*NBTyr. The nitro group of the leaving group forms a polar interaction only with the main chain of Ala70 in the hydrophobic environment. These comparisons indicate that two different mechanisms are achieved among the synthetase variants for the recognition of the same leaving group. Concerning *Mj*TyrRS, previous crystallographic studies (listed in Table S2) have shown that the substrate recognition mode can be altered by amino acid mutations at 19 sites. In combination with the results of this structural analysis, the finding in this present study is expected to provide a basis for the design of photoresponsive components other than the nitrobenzyl group.

### 2.3. Synthesis of Caged Proteins in a Cell-Free Protein Synthesis System

**Figure 3:**
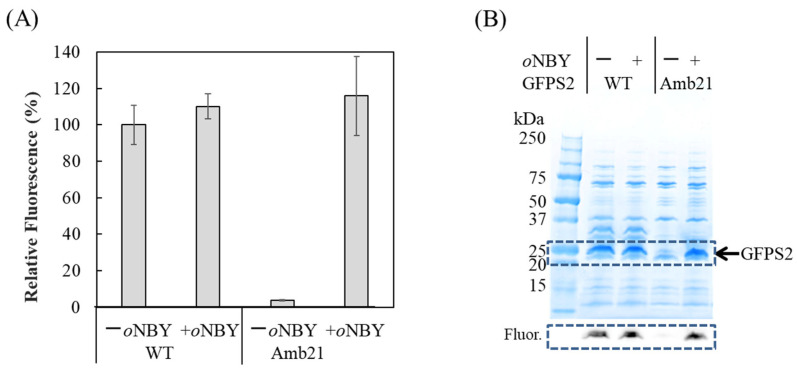
Cell-free protein synthesis of N11-GFPS2 with (+) or without (−) *o*-nitrobenzyl Tyr (*o*NBY) supplementation using the cell extract from the strain B95.deltaA. Wild-type GFPS2 is indicated as “WT” and GFPS2 with Y21amber mutation is indicated as “Amb21”. (**A**) Fluorescent measurement of GFP in the reaction solutions. The excitation and emission wavelengths are 485 nm and 535 nm, respectively. Data represent the mean ± standard deviation (SD) of four independent experiments; (**B**) SDS-PAGE analysis of fractions purified by Ni-NTA resin. The 10–20% polyacrylamide gel was stained with CBB after electrophoresis. A portion of a fluorescent image of the gel framed by the dashed line is shown in the lower panel. The fluorescent imaging was performed by Vilber Bio Imaging FUSION FX with 470 nm and 535 nm filters. Molecular weights of protein standards (BIO-RAD Cat. No. 1610363) are indicated.

We used a cell-free protein synthesis system with cell extracts from RFzero-iy, an *E. coli* strain deprived of the gene coding for the polypeptide release factor 1 gene (*prfA*) to facilitate efficient site-specific incorporation of nonnatural amino acids into proteins [20,21]. In this study, we employed cell extracts from the *E. coli* BL21 (DE3) derivative B95.delta A strain (B95.ΔA), in which the *prfA* gene was deleted and 95 selected UAG codons were replaced with UAA or UGA codons [22]. The large-scale preparation of cell extracts from B95.ΔA was performed as previously reported, except that the preincubation buffer was not used [23]. Efficient protein production with nonnatural amino acids was examined via *p*-benzoyl phenylalanine (*p*Bpa) incorporation into N11-GFPS2_Y21amber, in which the tyrosine codon at position 21 was replaced with the UAG codon (Supplementary Figure S2B). Similarly, with supplementation of 0.32 mg/mL *o*NBTyrRS and 0.375 mg/mL *o*NBTyr, we observed comparable amounts of products were synthesized from the N11-GFPS2_Y21amber gene (Figure 3). This cell-free protein synthesis is a strong option for the production of caged proteins, since medically relevant membrane proteins have been successfully synthesized using cell-free systems.

### 2.4. Crystallization of Caged Lysozyme and Decaging in the Crystal

**Figure 4:**
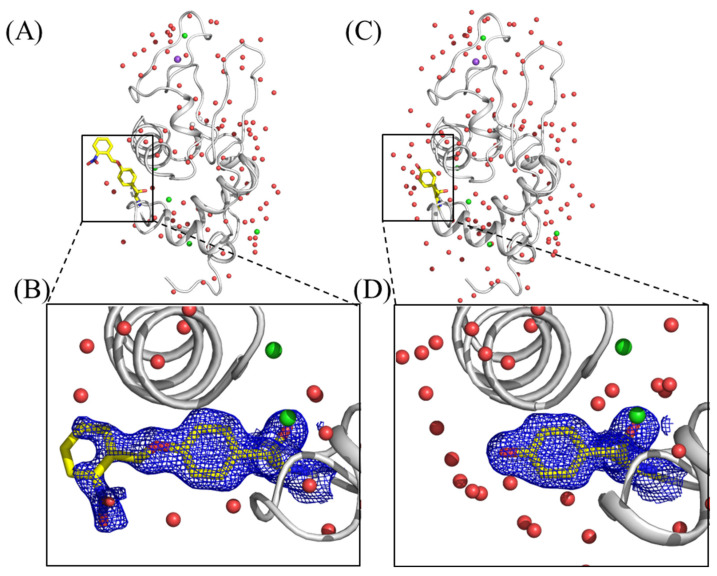
Crystal Structures of hen egg-white lysozyme (HEWL) with the incorporation of *o*NBTyr before (**A**) and after (**C**) photoactivation. Close-up views of the *o*NBTyr; (**B**) and decaged *o*NBTyr, Tyr; (**D**) at position 20 of HEWL are shown by stick yellow models. The |2F_o_ − F_c_| electron density maps (0.6 σ) around Tyr20 are depicted by blue mesh. Water molecules, sodium, and chloride atoms are shown by red, purple, and light green spheres, respectively.

To investigate the application of caged proteins to X-ray crystallographic study, we synthesized hen egg-white lysozyme (HEWL) with an *o*NBTyr substitution for Tyr20 (HEWL_Y20*o*NBTyr) by using the aforementioned *E. coli* cell-free protein synthesis system [24,25]. HEWL_Y20*o*NBTyr was purified from the cell-free reaction solution and crystallized using the sitting drop vapor diffusion method. The HEWL_Y20*o*NBTyr crystals yielded datasets at 1.44 Å resolution with completeness of 99.6% and a CC_1/2_ of 0.980. Model building and structure refinement were performed using Phenix (Table S1, PDB ID: 7YNU) [26]. The crystal structure of HEWL_Y20*o*NBTyr contained one protomer in the asymmetric unit (Figure 4A). Comparison of the HEWL_Y20*o*NBTyr structure with that of the wild-type lysozyme (PDB ID: 3WUL) shows that these structures are almost the same (RMSD, 0.202 Å) [27]. We observed a continuous density from the tyrosine residue at position 20, in which the nitrobenzyl group could be unambiguously modeled (Figure 4B). The density corresponding to the distal part of *o*NBTyr appeared somewhat weak, and the occupancy for atoms of the nitrobenzyl group is relatively low (0.5–1.0). This could be caused by the structure flexibility, as with *o*NPTyr (PDB ID: 6rtz) [9]. The tyrosyl moiety of *o*NBTyr20 is stabilized by the stacking interactions between Arg21 and Lys96 and the hydrogen bonding with Ser100 (Figure S3). On the other hand, the nitrobenzyl group forms an electrostatic interaction with Arg21, but is exposed on the surface (Figure S3A). Furthermore, the molecular geometry at this position does not affect the crystal packing environment. Alternatively, light contamination during protein synthesis or purification may have caused slight damage to the caged structure. These results confirm that efficient cell-free production of lysozyme with *o*NBTyr does not occur with any nonspecific suppression of the UAG codon with natural amino acids in the cell-free synthesis reaction. Finally, the crystals were irradiated with 365 nm light for 5 min for decaging. The obtained crystal structure at a 1.4 Å resolution showed that *o*NBTyr efficiently degraded into Tyr (Figure 4C,D). The overall structure of decaged HEWL was almost identical to that of caged HEWL. The RMSD per residue between structures before and after UV irradiation was 0.13. The release of the nitrobenzyl group resulted in the entry of water molecules into space, whereas a single water molecule was held in the vicinity of the oxygen atom in tyrosine (Figure S4). These results demonstrate that enzymatic reactions and protein structural changes can be induced by light emission on protein crystals, opening doors to time-resolved serial femtosecond crystallography of proteins using X-ray Free Electron Laser (XFEL). Shortening the irradiation time can be achieved through the use of high-intensity lasers and the development of new caged structures that are more efficiently photoreactive than the nitrobenzyl group.

## 3. Materials and Methods

### 3.1. Strain, Chemicals, and Enzymes

*o*NBTyr was purchased from Accela ChemBio Inc (San Diego, CA, USA). Almost all chemicals, including isopropyl-ß-D-thiogalactoside (IPTG), dithiothreitol (DTT), Tryptone (Cat. No. 35640-95), Yeast Extract Dried (Cat. No. 15838-45), glutathione oxidized form (GSSG), and glutathione reduced form (GSH), were purchased from Nacalai Tesque Inc. (Kyoto, Japan). *E. coli* disulfide-bond isomerase C (DsbC) was purified basically according to the previous report [28]. His_6_-tagged enzymes, small ubiquitin-like modifier (SUMO) protease, and TEV protease were purified according to previous studies [29,30]. The *p*-benzoyl-L-phenylalanyl-tRNA synthetase (*p*BpaRS), a mutant *Mj*TyrRS (Y32G, E107S, D158T, I159S, and D286R) was cloned to the pET21b vector with a TEV protease site and His_6_ tag fused to the C terminus.

### 3.2. Construction of Plasmids Vectors for Protein Production

Codon-optimized gene encoding residues 19-147 of chicken egg white lysozyme (HEWL, UniProt ID: P00698) were chemically synthesized (Thermo Fisher Scientific, Waltham, MA, USA). The gene was N-terminally fused with a modified natural poly-histidine affinity tag (N11) and a SUMO protease recognition site and cloned into the plasmid vector pCR2.1-TOPO (Thermo Fisher Scientific, Waltham, MA, USA) [31]. The amber codon TAG was substituted for Tyr20 of HEWL by PCR mutagenesis method to construct pCR-HEWL-Y20amber.

*Methanocalodococcus jannaschii* tyrosyl-tRNA synthetase (*Mj*TyrRS, UniProt ID: Q57834) which evolved to recognize *o*NBTyr had mutations Y32G, L65G, F108E, D158S, L162E, and D286R (*o*NBTyrRS) [11]. For the construction of pET21a-*o*NBTyrRS-TEV-His_6_, the gene encoding *o*NBTyrRS was chemically synthesized (Thermo Fisher Scientific, Waltham, MA, USA) and inserted into the pET-21a vector using the In-Fusion HD Cloning kit (Clontech, Shiga, Japan).

DNA sequences of synthetic genes and primers used for PCR are listed in Supplemental Table S3.

### 3.3. Expression, Purification, and Crystallization of the oNBTyr-tRNA Synthetase

*E. coli* Rosetta2 (DE3) cells transformed with the plasmid pET21a-*o*NBTyrRS-TEV-His_6_ were grown at 37 °C in a 2 L Luria–Bertani (LB) medium. When the optical density at 600 nm (OD_600_) reached 0.7–0.8, IPTG was added at the final concentration of 0.3 mM and the cells were cultivated at 20 °C for an additional 24 h. Cells were harvested at 8000× *g* for 10 min at 4 °C.

The cell pellet was suspended in lysis buffer A [20 mM Tris-HCl (pH 8.0), 1 M NaCl, 20 mM imidazole, and 20% glycerol] at 10 mL per gram and lysed by sonication on ice. The lysate was centrifuged at 16,000× *g* at 4 °C for 30 min. The supernatant was applied to HisTrap HP column (Cytiva, Tokyo, Japan), washed with a 10-column volume of buffer A, and eluted with a linear gradient of imidazole (20–500 mM imidazole). The eluate was dialyzed against sterile buffer A. After cleavage of the His-tag by TEV protease (0.08 mg/mL) for 16 h at 4 °C, the cleaved tag and His-tagged TEV protease were removed via passage through the HisTrap column. The flow-through fractions were pooled and applied onto a HiPrep 26/10 Desalting (Sephadex G25) column (Cytiva, Tokyo, Japan), equilibrated in 20 mM Tris-HCl (pH 8.0) buffer containing 0.1 M NaCl, 10% glycerol, and 1 mM DTT. Further purification was performed by a HiTrap Q column (Cytiva, Tokyo, Japan) with a linear gradient of 0–0.3 M NaCl. Finally, the pooled *o*NBTyrRS was dialyzed against a storage buffer [20 mM Tris-HCl (pH 7.5), 1 mM MgCl2, 1 mM DTT, 200 mM KCl, and 25% glycerol] for 16 h at 4 °C. The purified *o*NBTyrRS was concentrated to approximately 15 mg/mL using an Amicon Ultra-centrifuge device 10-kDa MWCO (Millipore, Burlington, MA, USA).

Co-crystallization of *o*NBTyrRS with the *o*NBTyr was performed by vapor diffusion using sitting drops of the crystallization solution at 20 °C. A 2 µL droplet of 10 mg/mL protein solution containing 1.4 mg/mL of *o*NBTyr mixed with the same amount of reservoir solution was equilibrated against 0.5 mL reservoir solution [20% PEG300, 3% PEG8000, 10% glycerol, and 100 mM Tris-HCl (pH 8.0)].

### 3.4. Data Collection, Structure Determination, and Refinement of the oNBTyr-tRNA Synthetase

The datasets of *o*NBTyrRS with *o*NBTyr were collected on SPring-8 BL32XU using a wavelength of 1.0 Å with the EIGER X 9M [32]. The crystal size was evaluated using the automated data collection system ZOO, developed at SPring-8 BL32XU [33]. After dose estimation using the crystal size, a helical data collection scheme was used with a beam size of 15 × 10 μm and an oscillation step of 0.1° (total 360°, 3600 images). The collected images were processed every 300 images (corresponding to 30°) using KAMO [34] with the XDS program [35]. The structure was solved by molecular replacement using the Phaser program [36] in the Phenix suite [26], with wild-type *M. jannaschii* tyrosyl-tRNA synthetase without RNA (PDB ID: 1J1U) as the search model [12]. The refinement was conducted using the Phenix programs [26], and the structure was manually rebuilt with the Coot program [37]. Data collection and refinement statistics are presented in Supplemental Table S1.

### 3.5. Preparation of E. coli S30 Extract and Construction of Cell-Free Protein Synthesis System for Site-Specific Incorporation of Nonnatural Amino Acids

The *E. coli* BL21 (DE3) derivative B95. delta A strain (B95.ΔA) was transformed by the plasmid vector piodoTyrRS-MJR1-gent(pACYC), which harbors *Mj*TyrRS recognizes tRNA^Tyr^ possessing the anticodon CUA, and minor tRNAs [22]. The transformed cells grew efficiently at 30 °C in 20 L of “3xYT” medium (24 g Tryptone, 15 g yeast extract, and 7.5 g NaCl per liter) supplemented with 0.15 mg/mL iodo-Tyr and reached 3.5–4.0 OD_600_ within 6 h (Supplementary Figure S2A). Cultivation was performed in a fermenter (TS-MW-30, TAKASUGI MFG. Co., Ltd., Tokyo, Japan) under the control of temperature (30 °C), aeration (20 L/min), pressure (0.02 MPa—near atmospheric pressure), and agitation (220 rpm). Cells were harvested using a tangential flow filtration (TFF) system and centrifuged at 7500× *g* for 23 min at 4 °C [23]. Two hundred grams of the cells were harvested from the 20 L culture. Large-scale preparation of cell extracts from B95.ΔA was performed as previously reported, except that the preincubation buffer was not used [23].

Efficient protein production with nonnatural amino acids was examined by *p*-benzoyl phenylalanine (*p*Bpa) incorporation into N11-GFPS2_Y21amber, in which the tyrosine codon at position 21 was replaced with the UAG codon of N11-GFPS2 (GenBank: LC185343.1) (Supplemental Figure S2B). The *p*BpaRS-TEV-His_6_ was purified in a similar manner to *o*NBTyrRS but using only HisTrap columns (Cytiva, Tokyo, Japan). After cleavage of the His-tag by TEV protease (0.08 mg/mL) for 16 h at 4 °C, the cleaved tag and His-tagged TEV protease were removed via passage through the HisTrap column once more. The flow-through fractions were pooled and dialyzed against a storage buffer consisting of 20 mM Tris-HCl (pH 7.5), 1 mM MgCl_2_, 6 mM 2-ME, 40 mM KCl, and 50% glycerol. Approximately 11.4 g of cells yielded 115 mg of *p*BpaRS (23 mg/mL, 5 mL).

The small-scale dialysis-mode cell-free protein synthesis with a 30 μL reaction mixture was performed at 25 °C and shaken at 240 rpm for 4 h, as previously described [23,38]. The concentration of *p*Bpa was 1 mM both inside (reaction solution) and outside (feed solution). *p*BpaRS was added only to the reaction solution at a concentration of 0.1–0.3 mg/mL. The GFPS2 fluorescence at 535 nm was measured using a multi-label counter, ARVO SX (PerkinElmer, Waltham, MA, USA) with excitation at 485 nm.

### 3.6. A Site-Specific Introduction of oNBTyr to HEWL by a Cell-Free System and Its Purification

To incorporate *o*NBTyr at position 20 of HEWL, cell-free protein synthesis was performed in the same way as in the production of N11-GFPS2_Y21amber, except that pCR-HEWL-Y20amber (2 μg/mL) was used as a template and 0.32 mg/mL *o*NBTyrRS and 0.375 mg/mL *o*NBTyr were added. No reducing reagent (DTT) was used, but instead, 0.4 mg/mL *E. coli* DsbC, 1 mM GSSG, and 4 mM GSH were added to correctly form cysteine disulfide bonds under oxidative conditions. After 6 h of incubation at 25 °C, the reaction solution was centrifuged at 15,000× *g* for 10 min. The supernatant was applied to the HisTrap column (Cytiva, Tokyo, Japan) equilibrated with 20 mM Tris-HCl (pH 8.0) and 1 M NaCl, which was washed extensively with 20 mM imidazole in the buffer and eluted with 20 mM Tris-HCl buffer (pH 8.0) containing 0.5 M NaCl and 500 mM imidazole. The His-tag was cleaved with a final concentration of 0.4 mg/mL C-terminal His-tagged SUMO protease and dialyzed against 20 mM Tris-HCl buffer (pH 8.0), which contained 0.5 M NaCl and 20 mM imidazole, for 16 h at 4 °C. The cleaved tag and the His-tagged SUMO protease were removed via passage through the HisTrap column. The flow-through fractions were concentrated with a 3 kDa MWCO Amicon Ultra filter unit and applied to Superdex 200 10/300 column (Cytiva, Tokyo, Japan), equilibrated in Milli-Q-water. The resulting peak fractions were collected and concentrated again. We obtained over 1.0 mg of HEWL_Y20*o*NBTyr from the 54 mL reaction solution.

### 3.7. Crystallization, Data Collection, Structure Determination, and Refinement of HEWL_Y20oNBTyr

HEWL_Y20*o*NBTyr was crystallized using the sitting drop vapor diffusion method. Next, 2 μL of the protein solution (13.5 mg/mL) was mixed with an equal volume of the precipitant solution, composed of 100 mM Na acetate (pH 3.6), 8% PEG6000, and 28% NaCl. The crystallization plates were incubated at 20 °C in the dark.

Data collection, processing, and refinement are the same as in Section 3.4. The structure of wild-type lysozyme (PDB ID: 4YM8) was used as the search model. Before data collection of “decaged” HEWL_Y20*o*NBTyr, the crystal was illuminated by a LED lamp (OptoCode LED-EXTA, 365 nm) for five minutes at 20 °C. Data collection and refinement statistics are presented in Supplemental Table S1.

## 4. Conclusions

In this study, the crystal structure of *o*NBTyrRS-*o*NBTyr was determined. It provides a structural basis for the recognition of caged tyrosine, *o*NBTyr by mutant TyrRS. The *o*-nitrobenzyl group is commonly used as a photoremovable protecting group. However, the *o*-nitrobenzyl group has lower efficiency and slower kinetics in photoreaction than other caging groups [*p*-Hydroxyphenacy, 2-(2-nitrophenyl)ethyl, and (Coumarin-4-yl)methyl, 7-nitroindoline] [39]. To apply time-resolved serial femtosecond crystallography at the XFEL facility, the introduction of nonnatural amino acids using a more reactive photoremovable protecting group is required. The structural information obtained in this study and the structural comparison with similar caged-tyrosine structures will provide the basis for the development of a new caged amino acid introduction system.

In addition, we confirmed for the first time that the photoremovable protecting group was removed via light irradiation of protein crystals, in which *o*NBTyr was introduced by the *E. coli* cell-free synthesis system. The *E. coli* cell-free synthesis system is capable of synthesizing proteins that are difficult to produce in cellular systems, such as membrane proteins and highly toxic proteins. The synthesis reaction volume is smaller than that of the *E. coli* culture system, which has the advantage of reducing the number of expensive nonnatural amino acids, making it useful for a variety of applied research.

## Data Availability

The Protein Data Bank (PDB) entry codes were 7YNW for *o*NBTyrRS, 7YNU for HEWL_Y20*o*NBTyr, and 7YNV for “decaged” HEWL.

## Supplementary Materials

The following supporting information can be downloaded at: https://www.mdpi.com/article/10.3390/ijms231810399/s1. References [40,41,42,43,44,45,46,47,48,49,50,51,52,53,54,55] are cited in the supplementary materials.
