# Supplementary material for: Crystal Structure of an Archaeal Tyrosyl-tRNA Synthetase Bound to Photocaged L-Tyrosine and Its Potential Application to Time-Resolved X-ray Crystallography
Source: Int J Mol Sci. 2022 Sep 8;23(18):10399. doi: 10.3390/ijms231810399 (PMC9499402; doi:10.3390/ijms231810399)
Supplement: Supplementary file 1 [file ijms-23-10399-s001.zip › ijms-1877760-supplementary.pdf]

Supplementary Materials

Figures S1-4

Tables S1-S3

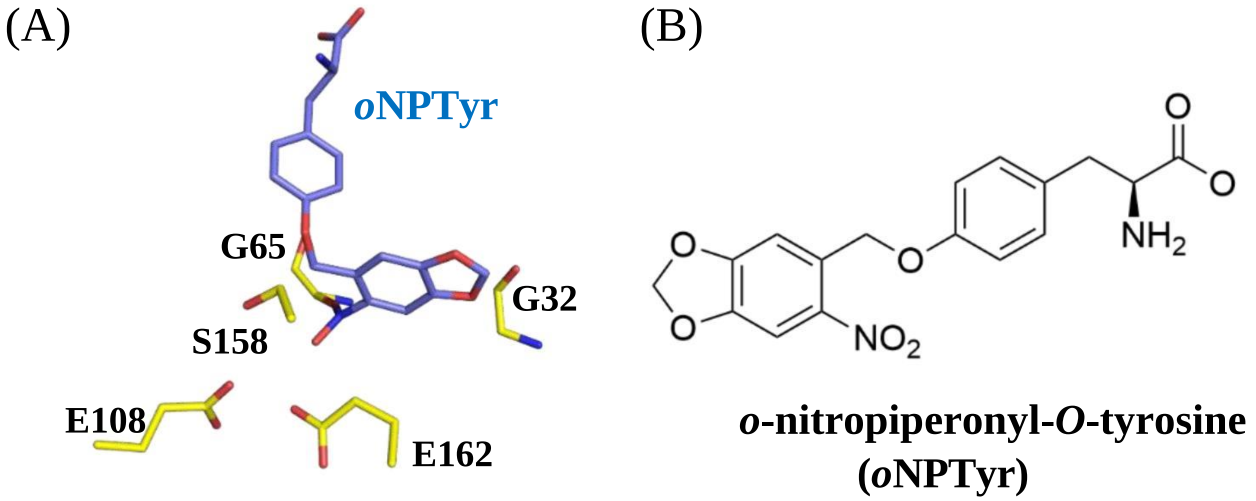

Figure S1. *o*NPTyr (light blue) modeled in the amino acid binding site of *o*NBTyrRS.

(A) The five mutated residues essential for recognizing *o*NBTyr are shown in yellow sticks. There is no structural clash with *o*NBTyrRS and the position of the *o*-nitrobenzyl group is the same as that of *o*NBTyr shown in Figure 1C.

(B) Chemical structure of *o*NPTyr.

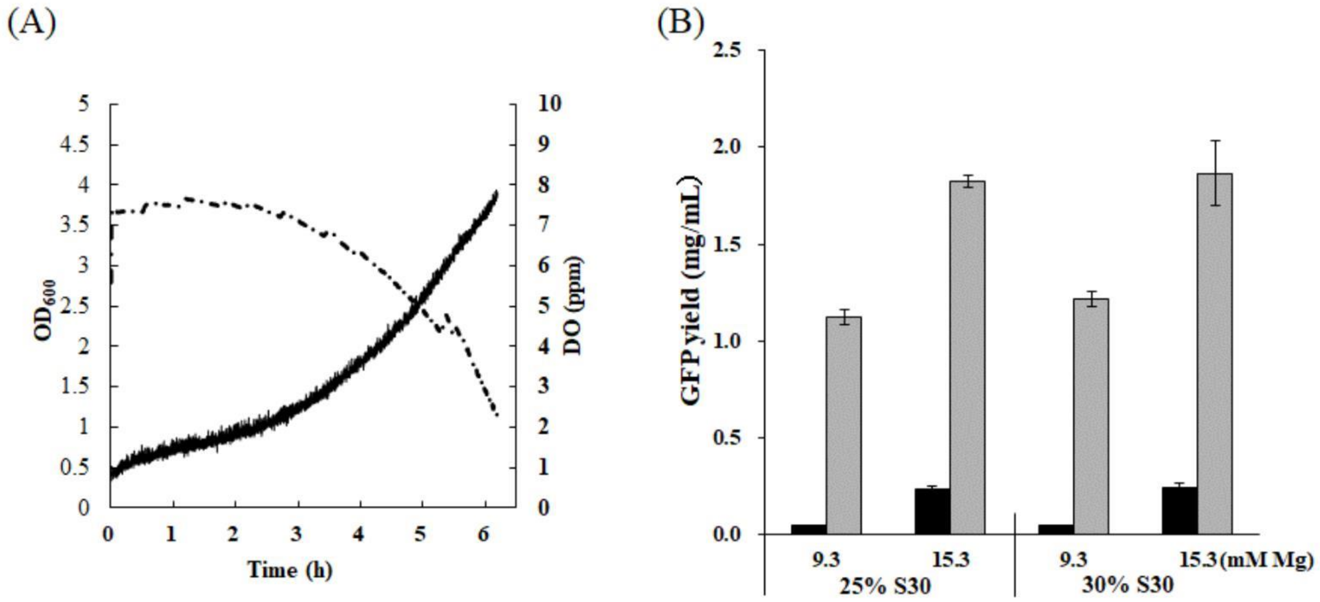

Figure S2. Preparation of cell-free protein synthesis system for site-specific incorporation of nonnatural amino acids.

(A) Continuous measurements of optical density at 600 nm (OD_{600}) and dissolved oxygen concentration (DO, dashed line) during culture of the *E. coli* strain B95. delta A transformed with the plasmid harboring iodoTyrRS and the cognate tRNA [22]. (B) Examination of concentrations of S30 extract and Mg acetate for production of N11-GFPS2 with Y21amber mutation with (gray) or without (black) *p*-benzoyl-Phe (*p*Bpa). Data represent the mean± standard deviation (s.d.) of three independent experiments.

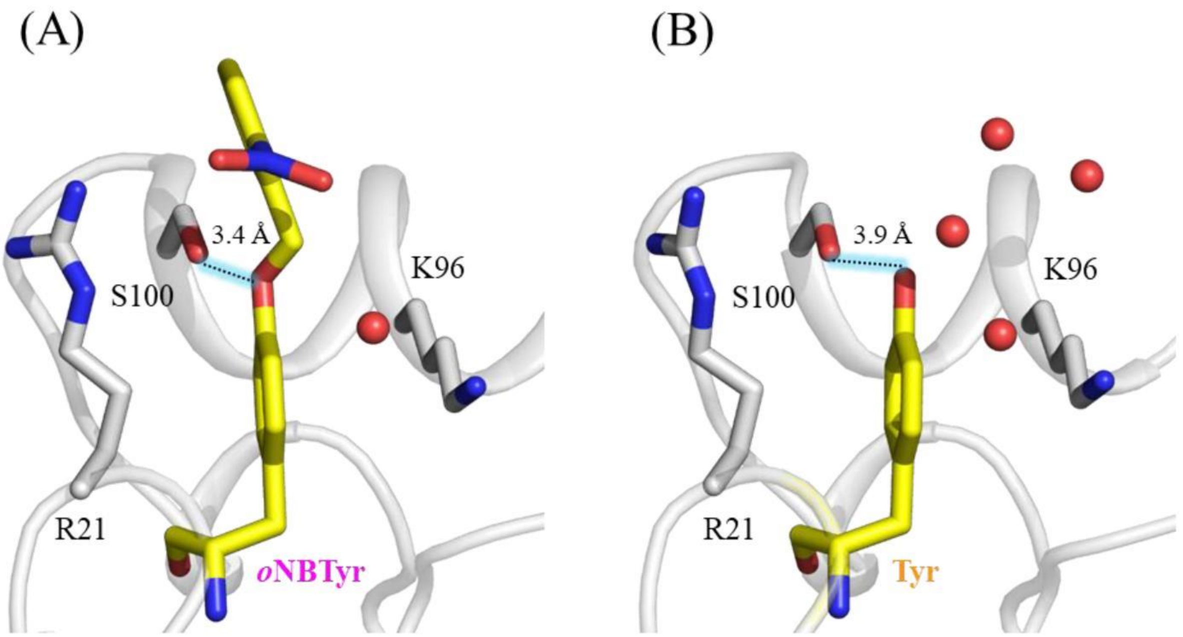

Supplemental Figure S3. Close-up views around position 20 of oNBTyr-incorporated HEWL in the dark state (A) and the light state (B).

(A) The structure of *o*NBTyr at position 20 and neighbor amino acids of the dark state lysozyme. The tyrosyl moiety of *o*BNTyr20 forms the stacking interaction between Arg21 and Lys96 and the hydrogen bond interaction between the oxygen atom and Ser100 at a distance of 3.4 Å. The nitrobenzyl group forms an electrostatic interaction with Arg21 at a distance of 4.3 Å. (B) The structure of Tyr at position 20 and neighbor amino acids of the photolyzed lysozyme. The distance between the oxygen atom and Ser100 is 3.9 Å. Water molecules are shown in red spheres.

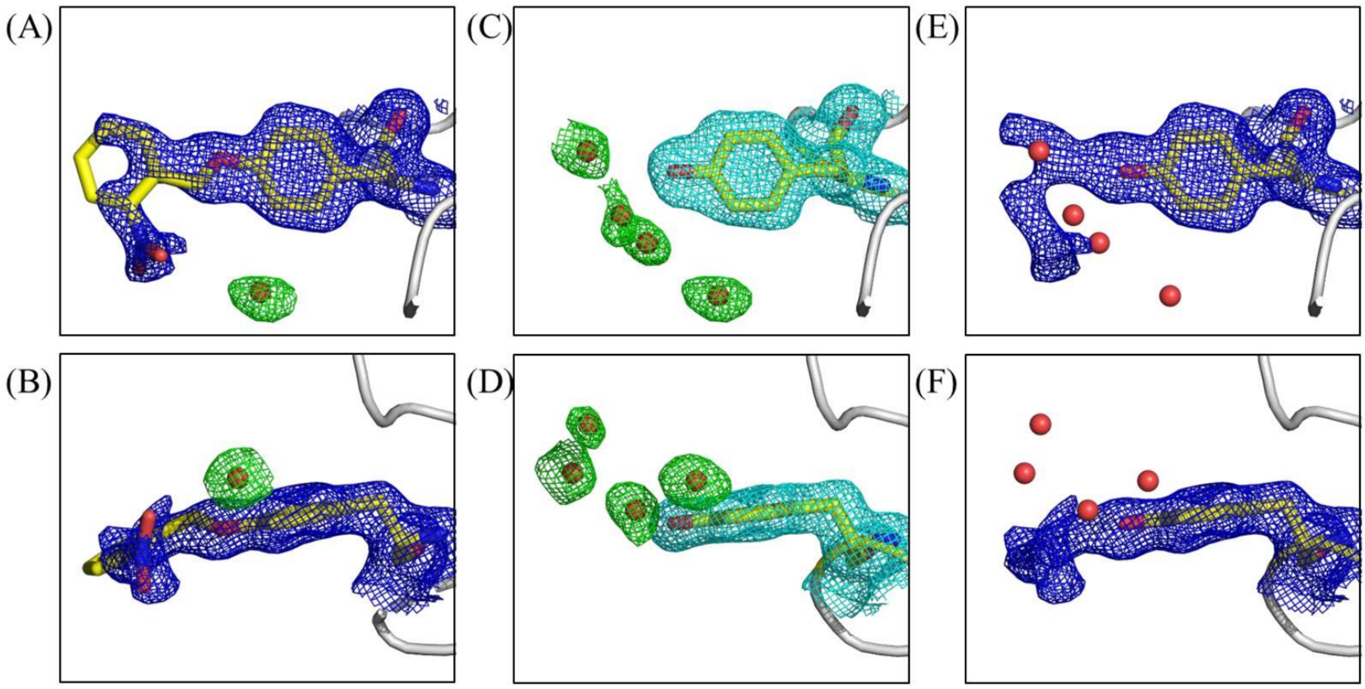

Supplemental Figure S4. Close-up views of the Tyr at position 20 of HEWL.

(A and B) The $|2F_o - F_c|$ electron density map (0.6σ) of the dark state lysozyme structure is represented by a blue mesh corresponding to ONBTyr at position 20 and a green mesh corresponding to water molecules within 5.1 Å around *o*NBTyr20. (C and D) The $|2F_o - F_c|$ electron density map (0.6σ) of the photolyzed lysozyme structure is depicted by a cyan mesh corresponding to Tyr20 and a green mesh corresponding to water molecules within 5.1 Å around Tyr20. (E and F) Superposition of the $|2F_o - F_c|$ electron density map (0.6σ) in the dark state (blue mesh) and the photolyzed lysozyme structure with Tyr20 (yellow sticks). Water molecules are shown in red. The upper panels were rotated 90° and shown in their respective lower panels.

Table S1: Crystallographic data and refinement statistics.

	Lysozyme		ONBTyrRS
	Dark	Light	Dark
PDB ID	7YNU	7YNV	7YNW
Data collection			
Resolution range	35.6-1.44 (1.49-1.44)	39.1-1.40 (1.44-1.39)	41.6-2.8 (2.89-2.79)
Space group	$P4_32_12$	$P4_32_12$	$P6_522$
Unit cell (Å)	a=79.7, b=79.7, c=37.1	a=78.3, b=78.3, c=37.4	a=83.5, b=83.5, c=488.7
Unit cell (˚)	α=90, β=90, γ=90	α=90, β=90, γ=90	α=90, β=90, γ=120
Number of total reflections	350464	526454	968582
Number of unique reflections	22080	23827	26487
Multiplicity	15.9 (15.0)	22.1 (16.4)	36.6 (37.2)
Completeness (%)	99.6 (100)	99.5 (95.1)	98.6 (98.5)
I/σ(I)	5.63 (0.91)	23.1 (6.1)	20.5 (0.7)
R_{merge}(%)	22.3 (96.2)	12.1 (40.4)	12.9 (506.8)
$CC_{1/2}$	1.0 (0.68)	1.0 (0.95)	1.0 (0.93)
Refinement			
R_{work} / R_{free} (%)	19.1/22.3	17.4/19.2	26.1/29.8
r.m.s.d. bond lengths (Å)	0.007	0.004	0.003
r.m.s.d. bond angles (˚)	0.89	0.64	0.589
Ramachandran plot			
Favored	98.4	98.4	97.7
Allowed	1.6	1.6	2.3
Disallowed	0	0	0
Average B factor ($Å^2$)	26	21	136.8
Protein	24.9	19.4	137.3
water molecules	34.7	32.8	116.8

Supplementary Table S2. Previously published structures of *Mj*TyrRS variants, mutated positions, and substrates (ligand)

Ligand	Positions of Mutated Residues																			PDB ID
Tyr (wild type)	M6	Y32	I63	L65	H70	Q75	E107	F108	Q109	Y114	Q155	D158	I159	Y161	L162	V164	A167	A180	C190	D286	1J1U [12]
*o*NBTyr		Gly		Gly				Glu				Ser			Glu					Arg	this work
4-(trimethylsilyl)-Phe		His	Gly	Val	Gln							Gly	Gly			Gly					7CKG [40]
3-nitro-Tyr		His			Thr							His	Ala		Arg						6WRK [41]
3-nitro-Tyr		His			Ala							Ser	Ala		Arg						6WRN [41]
3-nitro-Tyr		His			Cys							Cys	Ala		Arg						6WRQ [41]
3-nitro-Tyr		His			Ala							Cys	Ala		Arg						6WRT [41]
4-Borono-Phe	Leu	Ser		Ala	Met							Ser			Glu					Arg	5N5U [42]
β-1-azulenyl-Ala [1]		Gly		Trp	Gly			His	Asn			Ala			Asn					Arg	5NSF [43]
4-carboxymethyl-Phe		Ser		Ala				Lys	His			Gly			Lys						5U36 [44]
m-*o*NB-DOPA [2]		Ala		Ile	Ala				Ala			Ala	Ala		Met		Asn	Gln		Arg	5L7P [8]
3,5-dichloro-Tyr		Leu		Ile	Gly			Ile	Leu	Gly		Ser			Met						4NX2 [45]
Tco-amF [3]		Gly		Glu				Trp	Met			Ser			Lys						4PBT [46]
BibaF [4]		Gly		Glu				Trp	Met			Ser			Lys						4PBR [46]
BibaF [4]		Gly		Glu								Gly	Cys								4PBS [46]
m-nitro-Tyr		His			Gly							Ser	Ala		Arg						4NDA[47]
3,5-difluoro-Tyr		Arg		Tyr	Gly			Asn	Cys			Asn			Ser						4HJX [48]
4-cyano-Phe		Leu		Val				Trp	Met			Gly	Ala								3QE4 [49]
4-cyano-Phe		His			Cys	Asn						Ser	Ala		Arg						4ND6 [47]
4-cyano-Phe		His			Cys							Ser	Ala		Arg						4ND7 [47]
p-(2-tetrazolyl)-Phe		Leu		Ile					Met			Gly			Val	Gly					3N2Y [50]
3-iodo-L-Tyr					Ala							Thr	Ser							Tyr	2ZP1
4-(2,2,2-trifluoroethyl)-Phe		Ile			Phe		Ser		Met			Pro	Leu		Glu						3D6V [51]
p-trifluoromethyldiaziridinyl-Phe[5]		Ile			Phe		Ser		Met			Pro	Leu		Glu						3D6U [51]
3-(2,2'-bipyridin-5-yl)-Ala		Gly		Tyr	Ala						Glu	Gly	Trp		Ser						2PXH [52]
p-(benzoyl)-Phe		Gly					Pro					Thr	Ser	Arg					Met		2HGZ [53]
4-bromo-Phe		Leu					Ser					Pro	Leu		Glu						2AG6 [54]
β-(2-naphthyl)-Ala		Leu					Ser					Pro	Ala		Gln		Val				1ZH0 [54]
4-acetyl-Phe		Leu										Gly	Cys		Arg						1ZH6 [55]

[1] *β*-(1-azulenyl)-l-Ala: (2~S)-2-azanyl-3-(2,6-dihydroazulen-1-yl)-propanoic acid

[2] *m*-*o*NB-DOPA: (2~S)-2-azanyl-3-{3-[(2-nitrophenyl)methoxy]-4-oxidanyl-phenyl}-propanoic acid

[3] Tco-amF: *p*-trans-cyclooctene-amido-Phe: 4-{[(1R,4E)-cyclooct-4-en-1-ylcarbonyl]amino}-phenylalanine

[4] BibaF: *p*-2-bromoisobutyramido-Phe: 4-[(2-bromo-2-methylpropanoyl)amino]-phenylalanine

[5] *p*-trifluoromethyldiazirinyl-Phe: 4-[3-(trifluoromethyl)-diaziridin-3-yl]-phenylalanine

Table S3. DNA sequences of codon-optimized synthetic genes and primers

Name and description/ Sequence
A synthetic gene for *o*NBTyrRS with a TEV protease site and His_6-tag at the C-terminus
ATGGACGAATTTGAAATGATAAAGAGAAACACATCTGAAATTATCAGCGAGGAAGAGTTAAGAGAGGTTTTAAAAAAAGATGAAAAATCTGCT<br>GGTATAGGTTTTGAACCAAGTGGTAAAATACATTTAGGGCATTATCTCCAAATAAAAAAGATGATTGATTTACAAAATGCTGGATTTGATATAATTA<br>TAGGTTTGGCTGATTTACACGCCTATTTAAACCAGAAAGGAGAGTTGGATGAGATTAGAAAAATAGGAGATTATAACAAAAAAGTTTTTGAAGC<br>AATGGGGTTAAAGGCAAAATATGTTTATGGAAGTGAAGAACAGCTTGATAAGGATTATACACTGAATGTCTATAGATTGGCTTTAAAAACTACCTT<br>AAAAAGAGCAAGAAGGAGTATGGAACTTATAGCAAGAGAGGATGAAAATCCAAAGGTTGCTGAAGTTATCTATCCAATAATGCAGGTTAATTCT<br>ATTCATTATGAAGGCGTTGATGTTGCAGTTGGAGGGATGGAGCAGAGAAAAATACACATGTTAGCAAGGGAGCTTTTACCAAAAAAGGTTGTT<br>TGTATTCACAACCCTGTCTTAACGGGTTTGGATGGAGAAGGAAAGATGAGTTCTTCAAAAGGGAATTTTATAGCTGTTGATGACTCTCCAGAAG<br>AGATTAGGGCTAAGATAAAGAAAGCATACTGCCCAGCTGGAGTTGTTGAAGGAAATCCAATAATGGAGATAGCTAAATACTTCCTTGAATATCCT<br>TTAACCATAAAAAGGCCAGAAAAATTTGGTGGAGATTTGACAGTTAATAGCTATGAGGAGTTAGAGAGTTTATTTAAAAATAAGGAATTGCATCC<br>AATGCGTTTAAAAAATGCTGTAGCTGAAGAACTTATAAAGATTTTAGAGCCAATTAGAAAGAGATTATCGGATCCGGAAAACCTGTATTTTCAGG<br>GACTCGAGCACCACCACCACCACCACTGA
Primers for cloning to pET-21a with *Nde*I and *Xho*I sites (in *bold italic*)
Forward: TTTAAGAAGGAGATATA*CATATG*GACGAATTTGAAATGATAAAGAGAAACACATCTG
Reverse: GTGGTGGTGGTGGTGGTG*CTCGAG*TCCCTG
A synthetic gene for HEWL (19-147) with His-tag and SUMO-tag at the N-terminus
ATGAAAGATCATCTCATCCACAATCATCACAAACATGAGCACGCTCATGCCGAACACCTGGGGTCCGACTCAGAAGTCAATCAAGAAGCTAAGC<br>CAGAGGTCAAGCCAGAAGTCAAGCCTGAGACTCACATCAATTTAAAGGTGTCCGATGGATCTTCAGAGATCTTCTTCAAGATCAAAAAGACCAC<br>TCCTTTAAGAAGGCTGATGGAAGCGTTCGCTAAAAGACAGGGTAAGGAAATGGACTCCTTAAGATTCTTGTACGACGGTATTAGAATTCAAGCT<br>GATCAGACCCCTGAAGATTTGGACATGGAGGATAACGATATTATTGAGGCTCACAGAGAACAGATTGGTGGAAAAGTTTTTGGTCGTTGTGAAC<br>TGGCAGCAGCAATGAAACGTCATGGTCTGGATAACTATCGTGGTTATAGCTTAGGTAATTGGGTTTGCGTTGCCAAATTTGAGAGCAATTTCAAT<br>ACCCAGGCAACCAATCGTAATACCGATGGTAGCACCGATTATGGTATTCTGCAGATTAATAGCCGTTGGTGGTGTAATGATGGTCGTACACCGGG<br>TAGCCGTAATCTGTGTAATATTCCGTGTAGCGCACTGCTGAGCAGCGATATTACCGCAAGCGTTAATTGTGCCAAAAAAATCGTGAGTGATGGTA<br>ATGGTATGAATGCATGGGTTGCATGGCGTAATCGTTGTAAAGGCACCGATGTTCAGGCATGGATTCGTGGTTGTCGTCTGTAATGA
Primers for the introduction of TAG codon (in *bold italic*) at Tyr20 of HWEL
Forward: CTGGATAAC*TAG*CGTGGTTATAGCTTAGGTAATTGGGTT
Reverse: TATAACCACGC*TA*GTTATCCAGACCATGACGTTTCATTG
